# Genetic Basis of Multiple Antibiotic Resistance of Pathogenic *Escherichia coli* Strains Isolated from Livestock Complexes in Krasnodar Krai, Russia

**DOI:** 10.3390/ijms27010305

**Published:** 2025-12-27

**Authors:** Alexander Tishchenko, Mikhail Shumkov, Elizaveta Kazakova, Irina Tarasova, Dmitry Karpov, Sergey Kopyltsov, Anna Goncharenko

**Affiliations:** 1 Federal State Budgetary Educational Institution of Higher Education “Kuban State Agrarian University named after I.T. Trubilin”, 350044 Krasnodar, Russia; 2 Bach Institute of Biochemistry, Fundamentals of Biotechnology Federal Research Center, Russian Academy of Sciences, 119071 Moscow, Russia; 3 V.L. Talrose Institute for Energy Problems of Chemical Physics, N.N. Semenov Federal Research Center for Chemical Physics, Russian Academy of Sciences, 119334 Moscow, Russia; 4 Engelhardt Institute of Molecular Biology Russian Academy of Sciences, 119991 Moscow, Russia

**Keywords:** *Escherichia coli*, colibacillosis, escherichiosis, antibiotic resistance, virulence-associated genes

## Abstract

Antimicrobial resistance is a serious problem in veterinary medicine worldwide. Given that farm animals are considered to be among the sources of infectious disease pathogens in humans, studying the genetic diversity of pathogenic microorganisms isolated from them and identifying strains that are potentially pathogenic to humans is a primary research task nowadays. The current paper provides a comprehensive characterization of 4 *Escherichia coli* strains that caused colibacillosis in farm animals. Along with a description of morphological and biochemical characteristics, data on antibiotic sensitivity are presented, and a multiple antibiotic resistance index is calculated. In order to identify the genetic basis of antimicrobial resistance (AMR), whole-genome sequencing was performed, followed by phylogenetic analysis and identification of antibiotic resistance genes. It is shown that the studied strains possess genetic determinants of virtually all major molecular mechanisms of antibiotic resistance, a significant number of which are phenotypically expressed. The pronounced potential for acquiring new variants of resistance, in combination with the strains’ virulence potential, determines the etiological significance of the *E. coli* isolates in relation to both farm animals and human beings. A comparison of the results obtained with other researchers’ data indicates a coincidence in the trends of the multiple antibiotic-resistant strains spread in different countries and points to the need for the development and implementation of a unified global strategy to combat AMR.

## 1. Introduction

Antimicrobial resistance is a serious and growing problem in veterinary medicine worldwide. This is due to the global past usage of antibiotics for virtually any pathology control (both for therapeutic and prophylactic purposes), as well as for the increase in animal growth rates [1]. For a long time, antibiotics were used in agriculture with virtually no restrictions. Even now, their consumption in productive animals accounts for 70% of the total volume produced for veterinary use [1], and livestock complexes (especially, those huge) appear to be potential “hot spots” for the emergence and spread of antibiotic-resistant forms of bacteria [2,3].

Of the entire *Enterobacteriaceae* family, *E. coli* is the most common etiological agent of human and animal diseases, but at the same time, it is one of the most antibiotic-sensitive ones [4]. However, like other Gram-negative bacteria, this microorganism is resistant to hydrophobic antibiotics (such as macrolides, novobiocin, rifampicin, actinomycin D, and fusidic acid), that is associated with the low permeability of the bacterial outer membrane to fat-soluble substances. In addition, *E. coli* can easily acquire resistance to drugs to which it was previously highly sensitive (e.g., aminoglycosides, β-lactams, chloramphenicol, sulfonamides, tetracycline, nitrofurans, and fluoroquinolones) [4,5,6,7,8].

Of particular concern is *E. coli* multidrug resistance, which is defined as resistance to three or more classes of antimicrobial drugs [9,10]. Multidrug-resistant strains arise primarily due to the widespread distribution of genes located on mobile genetic elements, including plasmids, integrons, and transposons. In addition, the combination of these genes with resistance genes encoded on the chromosome can lead to the emergence of bacteria that are resistant to all major classes of available antimicrobial drugs [11]. Such multidrug-resistant bacteria are even found in healthy animals. In particular, in a study by Dimitrova et al. [10] found that 87.5% of non-pathogenic *E. coli* isolates from the feces of healthy pigs being fattened and from pig manure in lagoons were resistant to amoxicillin, ampicillin, tetracycline, chloramphenicol, trimethoprim/sulfamethoxazole, doxycycline hydrochloride, and nalidixic acid, and some also harbored broad-spectrum β-lactamase genes. Importantly, antibiotic resistance genes in enterotoxigenic *E. coli* can be located on the same conjugative plasmid as enterotoxin genes [12], which makes such strains yet more concerning.

*E. coli* can cause not only diseases of the digestive system, but also generalized systemic infections [13], which poses a particular danger in the case of multidrug-resistant strains of bacteria. The situation is exacerbated by the possibility of direct transmission of pathogens from animals to humans and vice versa. Through organic fertilizers and farm wastes, bacteria also enter the environment [14], where antibiotic resistance genes can be transferred horizontally to other microorganisms that were previously sensitive to antibacterial drugs.

This paper describes *E. coli* strains isolated in livestock complexes of Krasnodar krai (Russia) from three piglets and a calf with escherichiosis. It is primarily aimed at a detailed investigation of the strains’ antibiotic resistance features and the genetic basis of found insusceptibility phenotypes. The biochemical characteristics and phylogenetic analysis results are also presented and a comparison of the findings obtained with other researchers’ data is given.

## 2. Results

### 2.1. Phenotypic Characteristics of E. coli Strains

The strains studied were isolated in 2002 and 2003. At that time, they were identified as *E coli*, tested for their serotype and toxin production. Then they were deposited at −80 °C in the collection of the Kuban State Agrarian University, Russia. Every 5 years, the strains were tested for viability and pathogenicity in a white mouse infection model and demonstrated the retaining of their pathogenicity over the years. The biochemical characterization of the strains, their antibiotic susceptibility testing and whole-genome sequencing were performed in 2022–2025 with the newly grown cultures obtained with museum cultures used as an inoculum.

All strains obtained were non-spore-forming Gram-negative motile bacilli 0.5–1.0 × 1.5–3 μm in size. On LB agar, the isolates grew in a stable S-form, forming flat-convex, smooth, moist, shiny colonies with smooth edges, 2–4 mm in diameter, which were transparent and had a honey or greenish tint when viewed in transmitted light. On Endo medium, the colonies were raspberry-colored with a bronze tint. When inoculated in LB broth, visible turbidity of the medium appeared after 4–5 h of cultivation at 37–38 °C. After 14–16 h, the turbidity became abundant, a loose, easily broken sediment formed at the bottom of the cultivation tube, and a wall-adherent ring or film formed on the surface of the broth.

The morphophysiological and immunological characteristics of the isolated strains are described in Table 1. Biochemical characteristics are given in Table 2.

### 2.2. Sensitivity to Antibiotics

The determination of antibiotic resistance in the isolated strains showed their resistance to a wide range of antibacterial drugs (Table 3). Of the 40 antibiotics tested, belonging to 16 groups, all strains remained sensitive only to inhibitor-protected β-lactams (ticarcillin/clavulanic acid), some carbapenems (meropenem), and third-generation cephalosporins (cefotaxime). *E. coli* 533, 923, and 933 were also sensitive to sulfonamides (trimethoprim/sulfamethoxazole), *E. coli* 533, 546, and 923 to yet another cephalosporin (cefoperazone), *E. coli* 546, 923, and 933 to one of the fluoroquinolones (enrofloxacin). *E. coli* 546 and 923 could be also killed by aminoglycosides (amikacin). The *E. coli* 546 strain was characterized by the widest spectrum of antibiotic susceptibility and could be killed by ceftazidime (third-generation cephalosporin), imipenem (carbapenem), and ofloxacin (fluoroquinolone) as well.

The multiple antibiotic resistance (MAR) index was also defined as a tool for assessing risks to health and well-being. This index is useful for tracking the spread of bacterial resistance in a given population where resistance to more than three antibiotics is observed [15]. The multiple antibiotic resistance index calculated (Table 4) was 0.775 (*E. coli* 546), 0.825 (*E. coli* 923), and 0.875 (*E. coli* 533 and 933).

### 2.3. Sequencing of Complete Genomes of E. coli Strains

In order to understand the genetic basis of the observed phenotypic traits, whole-genome sequencing of isolated *E. coli* strains was performed using Illumina technology. General statistics on genome reads, assembly, and annotation are presented in Table 5.

Analysis using the Quast program (version 5.2.0, [16]), which evaluates the quality of genome assembly, showed that the sequencing and initial data processing yielded between 145 and 347 contigs (that were longer than 1000 bp) with a total length ranging from 5,042,042 to 5,130,089 bp. N50 varied from 16,921 to 111,514, and L50 varied from 16 to 92. Genome coverage was in the range from 12.8x to 54.1x. The GC-pair content was very close in different isolates and varied from 50.24% to 50.61%. According to the BUSCO program (version 5.5.0, [17]), the completeness of the genome assembly of the strain, estimated by the characteristic genomes of the *Enterobacterales* order, was at least 99.9% for the analyzed strains. The number of impurities according to the MiGA online server [18] ranged from 0 to 6.6%. Thus, the quality of the sequenced genomes of the analyzed strains was satisfactory and allowed for further analysis.

Based on the results of the Prokka program, the genomes of the analyzed *E. coli* strains contained between 4661 and 5060 protein-coding genes, of which from 603 to 1028 were hypothetical, and between 81 and 92 were tRNA genes.

### 2.4. Phylogenetic Position of the Isolated E. coli Strains

Using draft genomes, a search was performed for the most similar *E. coli* strains and other related species. According to the data obtained (Figure S1), *E. coli* 533 was close to the MG1655 substrain of the K12 strain, which is widely used in laboratories. However, the distance between these strains was significant, which can be explained by the presence of additional antibiotic resistance and virulence-associated genes in *E. coli* 533. *E. coli* strain 546 was very close to *E. coli* O104:H4 str. 2011C-3493, isolated in 2011 in Georgia from a patient suffering from bloody diarrhea [19]. *E. coli* strains 923 and 933 were closely related to the human pathogenic *E. coli* strain UMN026 (O17:K52:H18) isolated from a woman with uncomplicated acute cystitis in 1999 in the USA (Minnesota) [20]. Now this strain is a representative of the *E. coli* clonal group A that is widely disseminated and causes drug-resistant urinary tract and other extraintestinal infections [21].

The Clermont phylotyping scheme [22] is a popular tool for classifying *E. coli*, and many *E. coli* strains have been previously classified using this scheme alone. Therefore, we used the EzClermont web application to phylotype the strains under consideration [23]. According to the results obtained, strains 533 and 546 appeared to belong to the relatively small C phylotype, while strains 923 and 933 belonged to the D Clermont phylotype.

### 2.5. Description of Antibiotic Resistance and Virulence-Associated Genes

Table 6 summarizes the results of genome analysis of the studied *E. coli* strains through the usage of several databases. In particular, it shows the number of virulence associated genes (VAGs) and genes associated with antibiotic resistance [24], transporter genes [25,26], and drug targets [27].

Next, we performed a detailed search for genes associated with antibiotic resistance mechanisms (Table 7) using the RGI service in the CARD database [28]. The results obtained clearly indicated that the strains under study possessed virtually all the main molecular mechanisms of protection against antibiotics [29], which fully corresponded to the broad profile of resistance of the strains to most of the antibiotics tested (Table 3 and Table 4). Comparison of the detected genes also showed that the genetic determinants of antibiotic resistance in the 4 analyzed *E. coli* strains were identical. The only difference was observed in the genome of *E. coli* strain 533, which additionally possessed the *tetA* tetracycline resistance gene.

Uncovering the functions of genes identified in the isolates (Table 7), we discovered those encoding enzymes that inactivate antibiotics (all the strains carried β-lactamase (*bla*) genes), which ensured the destruction of β-lactam antibiotics of the penicillin, cephalosporin, oxime-cephalosporin, and monobactam groups, as well as genes of various multidrug resistance pumps (AcrAB-TolC, EmrAB-TolC, MdtABC-TolC, MdtEF-TolC, etc.) with a regulatory operon (*marRAB*), which allowed a wide range of antibacterial drugs, disinfectants, and other xenobiotics to be removed out of the cell. In addition, a gene encoding protection of the antibiotic target (*bcrC*) and genes associated with reducing cell wall permeability by changing its charge (*gdpD* and *pgsA*) were found.

## 3. Discussion

A study of the genetic basis of antibiotic resistance in the analyzed *E. coli* strains revealed almost complete similarity between the strains in terms of the spectrum of resistance genes. At the same time, at the phenotypic level, the sensitivity of the strains to antibacterial drugs did not coincide (Table 3), which indicated the inactivity of some resistance genes.

In the discussion, we matched the genotypic (Table 7) and phenotypic (Table 3) characteristics of the strains under consideration and deduced which resistance determinants directly influence the strains insusceptibility to certain antibiotics. The AMR genes and potentially mutated target genes are given in the context of the identified phenotypes of the *E. coli* isolates.

### 3.1. Resistance to Aminoglycosides

Aminoglycosides are bactericidal antibiotics that inhibit protein synthesis by binding to the aminoacyl site of 16S rRNA in the 30S subunits of bacterial ribosomes [30]. In veterinary medicine, gentamicin, neomycin, and apramycin are currently the most commonly used antibiotics from this group [https://www.merckvetmanual.com/pharmacology/antibacterial-agents/aminoglycosides-use-in-animals, accessed 29 October 2025]. There are three main mechanisms known to cause bacterial resistance to aminoglycosides. These are (a) inactivation of the antibiotic by acetyl transferases, nucleotidyl transferases, and phosphotransferases; (b) reduction of the intracellular concentration of antimicrobial drugs by decreasing membrane permeability (limited influx) or activating the expression of efflux pumps (active efflux); (c) methylation of 16S rRNA [9]. The latter of these mechanisms—modification of the 16S rRNA site by ArmA, RmtA-H, and NmpA methyltransferases—has become the most serious threat to aminoglycoside antibiotics. Of particular concern is the 16S rRNA methyltransferase ArmA, which is often found together with carbapenemase-type β-lactamases, since their genes can be located on the same mobile genetic element [11,31].

The found resistance to aminoglycosides of the analyzed strains might be determined by mutations in the S12p gene (almost all the representatives of this class of antibiotics) or *gidB* gene (streptomycin), as well as by the action of multidrug efflux pumps (primarily, AcrAD-TolC). At the same time, in *E. coli* strains 546 and 923, the specific resistance mechanisms were most likely associated with S12p gene mutations, since these strains were insensitive to gentamicin, streptomycin, and tobramycin, but could be eradicated by exposure to amikacin.

### 3.2. Glycopeptides Resistance

Glycopeptides (vancomycin) are rarely used in veterinary medicine. In particular, in the United States, these antibiotics are completely banned for use as drugs for farm animals. This is because vancomycin is active against most Gram-positive aerobes and anaerobes, but is ineffective against Gram-negative bacteria due to its large molecule and low cell penetration efficiency [https://www.merckvetmanual.com/pharmacology/antibacterial-agents/glycopeptides-use-in-animals, accessed 29 October 2025]. The *E. coli* strains we studied were resistant to glycopeptides. It is highly probable that this was connected with cell wall permeability, not with the mutations in the *ddl* gene.

### 3.3. Macrolides, Rifampicin, and Fusidic Acid Resistance

The *E. coli* strains under consideration were also characterized by resistance to macrolides, rifampicin, and fusidic acid. Despite the different mechanisms of action and targets of these antibiotics, they are all characterized by the hydrophobicity of their molecules, which limits their ability to penetrate the periplasmic membrane of Gram-negative bacteria and leads to the formation of resistance. So, the mechanism of insusceptibility to these antibiotics was not connected with a specific protein, but with physico-chemical features of the cell wall, though the MacAB-TolC drug efflux pump genes were found in the genome.

### 3.4. Resistance to Tetracyclines

One of the most commonly used classes of antibiotics in veterinary medicine is tetracyclines. They penetrate bacterial cell wall by passive diffusion and inhibit protein synthesis by binding to the 30S ribosomal subunit [https://www.merckvetmanual.com/pharmacology/antibacterial-agents/tetracyclines-use-in-animals, accessed 29 October 2025]. Due to frequent use, resistance to tetracyclines is widespread among *E. coli* strains [1]. A total of nine efflux pump genes *tet(A–EGJLY)*, two genes whose products prevent ribosome binding *tet(MW)*, and the *tetX* gene encoding an oxidoreductase that inactivates tetracyclines were identified [11]. The *tetABC* genes are the most common ones in *E. coli* strains obtained from productive and non-productive animals [9].

The *E. coli* strains we isolated were insusceptible to tetracycline and doxycycline as expected, and their resistance appeared to be determined by the activity of efflux pumps. For *E. coli* strains 536, 923, and 933, they were EmrKY-TolC protein complexes primarily, as well as MdfA and AcrAB-TolC possibly. In addition, *E. coli* 533 possessed the TetA pump gene.

### 3.5. Phenicols Resistance

The use of phenicols in veterinary medicine is associated with non-fluorinated chloramphenicol and fluorinated florfenicol. The former, due to its pronounced toxicity, is prohibited for use in productive animals but is permitted for the treatment of non-productive individuals. Florfenicol is licensed for the treatment of bacterial infections in both productive and non-productive animals [11].

The mechanism of action of phenicols is based on their binding to the 50S subunit of the ribosome and inhibition of peptidyl transferase activity during protein synthesis [32]. Resistance to phenicols in *E. coli* of animal origin, as in the case of aminoglycosides, can be mediated by three main mechanisms: (a) enzymatic inactivation of non-fluorinated phenicols by chloramphenicol acetyltransferases encoded by *cat* genes; (b) active efflux of non-fluorinated phenicols (*cmlA* gene) or fluorinated and non-fluorinated phenicols (*floR* gene); (c) methylation of the target RNA site by rRNA methyltransferase encoded by the *cfr* gene, which confers resistance to five different classes of antimicrobial agents [33].

The *E. coli* strains analyzed showed resistance to both chloramphenicol and florfenicol. However, the observed phenotype appeared to be due to the activity of specific (Cmr) and/or non-specific (AcrAB-TolC and MdtM) efflux pumps exclusively.

### 3.6. Polypeptide Antibiotics Resistance

The *E. coli* strains under consideration were also characterized by resistance to polypeptide antibiotics (colistin or polymyxin E). The mechanism of colistin’s antibacterial activity is due to its interaction with the lipopolysaccharide (LPS) of the cell wall of Gram-negative bacteria. This leads to an increase in the permeability of the cell membrane, the release of cytoplasmic components into the environment, and the death of bacterial cells [34,35].

Colistin is widely used in veterinary medicine, mainly for the treatment or prevention of intestinal infections, especially intestinal infections in newborn calves, piglets, and poultry [1]. *E. coli* strains with acquired resistance to colistin are most commonly found among pathogenic strains isolated from pigs suffering from diarrhea [36]. Resistance to this drug is based on mutations responsible for modifying the composition of LPS. Until recently, resistance to polymyxin (colistin) was known to be caused by chromosomal mutations only, which prevented it from spreading to other bacteria. However, in a relatively recent study conducted in China, the first transmissible plasmid causing colistin resistance determined by the *mcr-1* gene was described in commensal *E. coli* cells isolated from productive animals [37]. This gene encodes the phosphoethanolamine transferase MCR-1, the production of which leads to modification of the lipid part of LPS and a 4–8-fold increase in the minimum inhibitory concentration of polymyxins [11]. Although the *mcr-1* gene was first discovered in *E. coli* strains, it can easily be transferred between different species of bacteria and has already been identified in *Salmonella*, *Shigella*, *Klebsiella*, and *Enterobacteriaceae* family bacteria circulating among both animals and humans. To date, 11 variants of the *mcr-1* gene have been identified, and 6 very similar genes, designated *mcr-2-7*, have been discovered [11]. Due to the possible transfer of polymyxin resistance genes from bacteria of animal origin to human circulating bacteria, recommendations have been developed to discontinue the use of colistin in animal husbandry [1].

In our study, all four *E. coli* strains tested showed resistance to polymyxin E (colistin), but no *mcr* genes were found in their genomes. This might either indicate the presence of some other, as yet unknown, variants of phosphoethanolamine transferases, the existence of an alternative mechanism of resistance to polypeptide antibiotics, or features of the cell wall that inhibited the action of colistin. The issue requires further studying.

### 3.7. Sulfonamides Resistance

Sulfonamides, which inhibit various stages of folic acid synthesis, are widely used synthetic antimicrobial agents. Trimethoprim acts on the same metabolic pathway. Trimethoprim and sulfonamides act bacteriostatically when used separately, but their combination leads to a synergistic bactericidal effect [38].

Resistance to sulfonamides and trimethoprim is usually associated with the substitution of sensitive folate biosynthesis enzymes by isoenzymes that are insensitive to these compounds, and the corresponding genes are often located on plasmids [9,11]. Specifically, resistance to sulfonamides in *E. coli* strains isolated from productive animals and companion animals is mediated by the *sul1*, *sul2*, or *sul3* genes. The first gene (*sul1*) is particularly widespread, as it is part of the 3′-conservative segment of class 1 integrons [11]. Resistance to trimethoprim is caused by numerous *dfr* genes, which, depending on their size and structure, are divided into two main groups: *dfrA* and *dfrB*. In *E. coli* of animal origin, *dfrA* genes are predominantly detected [11].

Among the strains we analyzed, only *E. coli* 546 was resistant to the combination of trimethoprim and sulfamethoxazole. Since *sul* genes were not found in the strains under consideration, insensitivity to this combination of drugs was obviously due to mutations in metabolic pathway genes (*dfr*, *folA*, and *folP*).

### 3.8. Resistance to Fluoroquinolones

*E. coli* resistance to fluoroquinolones is usually caused by changes in drug targets, i.e., mutations in the DNA gyrase and topoisomerase IV genes [39]. DNA gyrase is the only enzyme capable of introducing negative supercoils into DNA. The absence of negative supercoiling makes DNA replication and bacterial reproduction impossible. At the physiological level in a single cell, inhibition of DNA gyrase leads to rapid bacterial death due to the formation of multiple breaks of both DNA strands. Topoisomerase IV also affects the topology of double-stranded DNA, but, unlike DNA gyrase, it is responsible for decatenation—the separation of daughter DNA strands during cell division. In addition to the modification of target, resistance to fluoroquinolones may also be due to a decrease in the permeability of the outer membrane or the activation of drug efflux pumps [40]. At that, resistance genes can be localized on either chromosome or plasmids.

In recent years, there have been an increasing number of reports on fluoroquinolone-resistant bacterial strains. In addition to bacteria that are insensitive to the commonly used levofloxacin or ciprofloxacin, strains that are insensitive to enrofloxacin also appeared. Thus, in Brazilian farms, almost 30% of *E. coli* isolates that caused neonatal colibacillosis were found to be resistant to this antibiotic [41].

All four *E. coli* isolates we studied were resistant to levofloxacin, norfloxacin, ciprofloxacin, and, with the exception of *E. coli* 546, to ofloxacin. *E. coli* 533 was the only to be insensitive to enrofloxacin (Table 3). It had the highest multiple antibiotic resistance index like *E. coli* 933 as well (Table 4). The phenotypes detected could be based on both mutations in the *gyrA* gene and the activity of multidrug efflux pumps (AcrAB-TolC, AcrEF-TolC, EmrAB-TolC, MdtEF-TolC, and MdtM).

### 3.9. Resistance to β-Lactam Antibiotics

Multiple efflux pumps also appear to contribute significantly to the development of antibiotic resistance in the case of β-lactam antibiotics. The aforementioned protein complexes AcrAB-TolC, AcrEF-TolC, and MdtEF-TolC may be involved there. At the same time, the main mechanism of resistance to β-lactams is based on β-lactamase activity, with the most problematic enzymes of extended-spectrum β-lactamases (ESBL) or cephalosporinases (AmpC), which provide *E. coli* extended resistance to penicillins, aminopenicillins, and cephalosporins including their fourth-generation representatives [5,9].

The *E. coli* isolates of the current work harbored BlaEC family β-lactamases belonging to the extended-spectrum AmpC (ESAC) group. These enzymes are capable of cleaving both penicillins and extended-spectrum cephalosporins, with significantly increased MICs for ceftazidime and cefepime, and in some cases also carbapenem compounds [42,43]. Such broad substrate specificity was consistent with the experimental results obtained. At the same time, the spectrum of β-lactam antibiotics to which the isolates were resistant was not the same for different strains, indicating the presence of various ESAC in their cells. As well, it should be noted that BlaEC β-lactamases are generally not inhibited by clavulanic acid [44], which was confirmed by our data on sensitivity to a mixture of clavulanic acid and amoxicillin. At that, ticarcillin in the presence of clavulanic acid inhibited the growth of the isolates, which draws to the conclusion that the β-lactamases we were dealing with were unable to destroy this antibiotic.

Unfortunately, AmpC-type β-lactamases that are capable of hydrolyzing a wide range of β-lactam antibiotics (ESAC) are widespread currently. ESBL/AmpC-producing *E. coli* can be isolated not only from clinically ill animals, but also from healthy individuals and even from the environment [10].

There is little information on the prevalence of carbapenemases in *E. coli* of animal origin, which is due to the rare use of carbapenems (meropenem, imipenem) in the treatment of productive and non-productive animals. Meanwhile, *E. coli* isolates that were resistant to these β-lactams have already been detected in various countries (Germany, France, the USA, China, India, Algeria) and in various animals (pigs, cattle, dogs, cats, poultry, fish) [11,45]. The strains we studied were sensitive to meropenem. However, three of the four (75%) showed resistance to imipenem. Since various penicillins are widely used in veterinary medicine and they are the universal substrate for various β-lactamases (including carbapenemases), the spread of resistance to β-lactam antibiotics is expected to increase.

### 3.10. Some Other Antibiotic Groups Resistance

The *E. coli* isolates under consideration, in addition to the antibiotics already mentioned, were resistant to nitrofurans, oxazolidinones, amphotericin, and lincosamines. No specific determinants of resistance to these compounds were found in course of the genome analysis, suggesting that insensitivity to the antibiotics was due to the activity of multidrug efflux pumps. First and foremost, AcrAB-TolC pumps appeared to be involved. The MdfA, MdtL, and MdtM systems may also contribute to the resistance development with MdtM’s role in protection against lincosamide antibiotics experimentally confirmed [https://card.mcmaster.ca/ARO:3001214, accessed 29 October 2025].

Overall, the study of the isolates’ resistance to various antibiotics showed the multidrug resistance index (MAR) of these strains to range from 0.775 to 0.875, which is extremely high. MAR values above 0.2 indicate high-risk sources of biological distress, where multiple antimicrobial drugs should be often used to control disease. The high resistance of the strains studied may be associated with the misuse of antibiotics for therapeutic purposes or widespread antibiotic use as growth promoters in farm animals.

Unfortunately, the problem is globally significant. Thus, a study conducted in Italy to assess the trend in the development of resistance in pathogenic *E. coli* isolated from weaned piglets between 2002 and 2011 showed an increase in the number of isolates resistant to enrofloxacin (from 14.5% to 89.3%), flumectin (from 49.1% to 92.9%), cefquinome (from 3.8% to 44%), gentamicin (from 63.6% to 85.7%), apramycin (from 61.8% to 82.1%), and trimethoprim/sulfamethoxazole (from 75% to 89.3%) [46,47]. High levels of antibiotic resistance (50 to 100% to 5 or more drugs) were reported for *E. coli* isolated from sick pigs and calves in Belgium, Poland, Spain, Germany, France, and Sweden [1,48,49]. Significant spread of antibiotic resistance in *E. coli* isolates from piglets with post-weaning diarrhea has been found in Australia and Malaysia [50,51]. The development of multidrug resistance was also noted in a study of the sensitivity of pathogenic *E. coli* isolated from weaned piglets with diarrhea in Korea [52].

It should be especially emphasized that the studied strains retained their antibiotic resistance genes and pathogenicity islands through more than 20 years of storage in cryoconserved state. The observation is of high significance for veterinary microbiology and epidemiology since it indicates the possible preservation of such bacterial features in harsher environmental conditions providing sick animals and their waste products to be a potential source of pathogenic bacteria, which harbor a wide range of antibiotic resistance determinants being able to be preserved for ages.

## 4. Materials and Methods

### 4.1. Isolation and Biochemical Identification of E. coli Strains

The strains were isolated and identified in accordance with the bacteriological method recommended by Order No. 535 of 1985 on the unification of microbiological research methods. We also used the Methodological Guidelines for the Bacteriological Diagnosis of Colibacillosis (Escherichiosis) in Animals of the Department of Veterinary Medicine of the Ministry of Agriculture and Food of the Russian Federation dated 27 July 2000, No. 13-7-2/2117 and the Methodological Guidelines for the Bacteriological Diagnosis of Mixed Intestinal Infections in Young Animals Caused by Pathogenic Enterobacteria of the Department of Veterinary Medicine of the Ministry of Agriculture and Food of the Russian Federation No. 13-7-2/1759 dated 11 October 1999.

The collection of clinical samples was conducted before antibiotic therapy of sick animals. Samples were placed in sterile test tubes and inoculated in nutrient media within 4 h. Otherwise, preservative solutions were used: either phosphate buffer (pH = 8.0), or a 1:1 glycerol-saline (0.9% NaCl solution) mix.

The strains were isolated in 2002 and 2003. That time they were identified, tested for serotype and toxin production and then deposited as −80 °C frozen samples in the collection of the Department of Microbiology, Epizootology, and Virology of Kuban State Agrarian University, Russia. The conditions of cryoconservation were as follows. Bacterial cultures were grown in LB nutrient medium for 24 h. Then 1 mL of 1 × 10^9^ CFU/mL bacterial suspension was mixed with sucrose-gelatin cryoprotectant (final 10% sucrose and 2% gelatin). Every 5 years, the strains were tested for viability and pathogenicity in a white mouse infection model. The whole-genome sequencing of the isolates was conducted in 2022; their biochemical characterization and antibiotic resistance testing were performed in 2023 and 2025 correspondingly.

The isolated strains (Table 8) were verified using the ENTEROtest 16 and ENTEROtest 24 diagnostic test systems (Erba Lachema s.r.o., Brno, Czech Republic), which is a Russian-language version of Microb-2 (Microbiological Monitoring System Microb-2, SMMM-2).

### 4.2. Cultivation of Microorganisms

Cultivation of *E. coli* strains 533, 546, 923, and 933 was routinely carried out on LB Agar (Lennox, Laboratorios Conda, Madrid, Spain) nutrient medium. In the course of phenotypic characterization Endo agar, SMAC-agar (Sorbitol-MacCONKEY agar) and CAYE Broth (Merck, Darmstadt, Germany) were also used. 24 h cultures of each strain on solid media were obtained through incubation in a Binder FD53 thermostat (BINDER GmbH, Tuttlingen, Germany) at 37 °C. Liquid *E. coli* cultures were grown in Erlenmeyer flasks filled with LB nutrient broth in Biosan shaker-incubator (Biosan, Riga, Latvia) at 200 rpm. To prepare blood agar for the determination of the hemolytic activity, melted Hottinger nutrient agar cooled to 45–50 °C was mixed with defibrinated sheep blood (7% *v*/*v*).

### 4.3. Deposition in Databases

Genome sequencing and assembly were placed in the NCBI database under the general number BioProject PRJNA887444. Each bacterial strain was also registered under individual number (Table 9).

### 4.4. Isolation of Bacterial DNA

For DNA extraction, 2 mg of bacterial biomass was scraped down out from 12 h slant agar tubes. The samples were suspended in 0.5 mL of TE buffer and centrifuged for 2 min at 14,000 rpm. The supernatant was removed. DNA was isolated from the cell pellet using the DNeasy Blood & Tissue Kit (QIAGEN, Hilden, Germany) according to the manufacturer’s instructions. The amount of isolated DNA was assessed using a Qubit fluorimeter (ThermoFisher, Waltham, MA, USA).

### 4.5. Whole-Genome Sequencing

To determine the nucleotide sequences of the genomes of the *E. coli* strains under study, libraries were prepared using the DNA Preparation (M) Tagmentation Kit (Illumina Inc., San Diego, CA, USA). The required concentration of the libraries was selected in accordance with the Qubit fluorimeter readings. Whole-genome sequencing was performed using a MiSeq device (Illumina Inc., USA). The resulting fastqc files were used for de novo genome assembly using SPAdes v.3.15.3 [53] in the Unipro UGENE v.52.1 program [54]. The quality of genome assembly was evaluated using QUAST v.5.0.2 [16]. The resulting contigs were deposited in NCBI as read archives and genomes, after which their coding DNA sequences (CDS) were automatically annotated using the NCBI Prokaryotic Genome Annotation Pipeline (PGAP) [55].

### 4.6. Antibiotic Susceptibility Testing

Antibiotic susceptibility testing was performed in accordance with the recommendations of the Clinical and Laboratory Standards Institute [56] as well as following clinical guidelines “Determination of the susceptibility of microorganisms to antimicrobial agents” of the Interregional Association of Clinical Microbiology and Antimicrobial Chemotherapy (MACMAX, version 2024-02; EUCAST, Clinical Breakpoint Tables v. 15.0, valid from 1 January 2025).

Antibiotic sensitivity testing began with the preparation of an inoculum (a suspension of microorganisms). To do this, a pure culture of the tested strains of *E. coli* was spread onto a plate with a solid Mueller-Hinton nutrient medium (Himedia, Mumbai, India) and incubated for 18–24 h at 35 ± 2 °C. Several isolated colonies of the same morphotype were then selected. They were suspended in sterile saline, and the suspension was adjusted to 0.5 optical density according to McFarland turbidity standard using a Den-1 densitometer (Biosan). Then, using a sterile cotton swab, the standardized suspension of microorganisms was distributed over the surface of a Petri dish with Mueller-Hinton agar. Excessive inoculum was removed by pressing the swab against the walls of the test tube. The Petri dish was allowed to dry for 10–15 min at room temperature. Filter paper discs containing standard amounts of antibiotic substances were placed on the surface of the agar using sterile tweezers or an applicator. The discs were placed at a distance of at least 15–20 mm from each other and from the edge of the dish. The dishes were incubated upside down at 35 ± 2 °C for 16–20 h. When estimating the results, the diameter of the growth inhibition zone around the disc was measured in millimeters, including the diameter of the disc itself (6 mm). The result was interpreted according to clinical recommendation tables, assigning one of the following categories: S—susceptible (high probability of clinical efficacy at standard dosage); and R—Resistant (clinical efficacy is unlikely).

The following antimicrobial drugs were used: azithromycin (15 μg), amikacin (30 μg), amoxicillin (25 μg), amoxicillin/clavulanic acid (20/10 μg), ampicillin (10 μg), amphotericin (40 μg), benzylpenicillin (10 units), vancomycin (30 μg), gentamicin (10 μg), doxycycline (30 μg), imipenem (10 μg), carbenicillin (100 mcg), clarithromycin (15 mcg), clindamycin (2 mcg), colistin (300 units), levomycetin-chloramphenicol (30 mcg), levofloxacin (5 mcg), linezolid (30 μg), meropenem (10 μg), norfloxacin (10 μg), oxacillin (1 μg), ofloxacin (5 μg), rifampicin (5 μg), streptomycin (10 μg), tetracycline (30 mcg), ticarcillin/clavulanic acid (75/10 mcg), tylosin (15 mcg), tobramycin (10 mcg), trimethoprim/sulfamethoxazole (1.25/23.75 mcg), florfenicol (30 mcg), fusidic acid (10 mcg), furadonin (300 mcg), cefoperazone (75 mcg), cefotaxime (30 μg), ceftazidime (30 μg), ceftriaxone (30 μg), cefuroxime (30 μg), ciprofloxacin (5 μg), enrofloxacin (5 μg), erythromycin (15 μg).

The MAR (multiple antibiotic resistance) index was defined as the ratio of the number of antibiotics to which the tested *E. coli* strains were found to be resistant to the total number of antibiotics used to assess sensitivity. A MAR index greater than 0.2 indicates a biological environment at high risk of infection and antibiotic use.

### 4.7. Phenotypic Characterization of Pathogenicity Factors

To decipher if the *E. coli* cultures could produce LT and/or ST toxins, biological assays in mice were used [12]. Two different experimental approaches were applied: either the study of delayed hyperresponsiveness in the mouse paw edema test [57] or a test on suckling mice [58].

The ability of the studied strains to produce STX toxins was confirmed by ELISA using the commercial test system RIDASCREEN VEROTOXIN 1 and 2 SLT I/II (R-Biopharm AG, Germany) [59].

### 4.8. Bioinformatic Data Analysis

The quality of the assembly and annotation of the genome was assessed using BV-BRC [60] in Comprehensive genome analysis mode, as well as using the RAST tool kit [61]. The search for genes encoding antibiotic resistance factors and VAGs was performed using databases such as the Transporter Classification Database (TCDB) [62], the virulence factor library in PATRIC [26], the virulence factor database (VFDB) [63], the Therapeutic Target Database (TTD), DrugBank [64], and the comprehensive antibiotic resistance database (CARD) [24].

A phylogenetic analysis of the *E. coli* strains was conducted using the following strategy. First, a set of reference and representative genomes that were most similar to the query genome were identified by Mash/MinHash [65], using a pre-formed database provided by PATRIC. Secondly, PGFams [66] were selected from all the aforementioned genomes, including the query genome, and aligned using MUSCLE [67]. Subsequently, the nucleotides from the genomes were mapped to this alignment. Ultimately, the entirety of the data was consolidated into a data matrix, which was then subjected to analysis by RAxML [68]. The tree’s support values were generated with the assistance of fast bootstrapping [69].

## 5. Conclusions

Our results indicate that pathogenic *E. coli* strains isolated from sick farm animals often possess multiple drug resistance, allowing them to actively resist various antibiotics and chemotherapeutic drugs. On the one hand, this property can lead to veterinary infections that are extremely difficult to combat, and on the other hand, it provides a vast reservoir of resistance determinants to most families of antimicrobial drugs that are important for both animals and humans. Based on this, a necessary and essential procedure in the process of identifying clinical isolates of *E. coli* is the detection and careful continuous monitoring of their antibiotic resistance profile, and information on the dynamics of the spread of individual resistance mechanisms is also undoubtedly important for the formation of an antibacterial therapy strategy.

The fight against the ever-increasing resistance of *E. coli* and other bacteria to antimicrobial drugs currently lies mostly in the creation of new compositions of antibiotics and in the increase in their doses recommended for application. Ultimately, this only leads to the development of additional defense and resistance mechanisms in microorganisms. Since the *E. coli* strains studied here, isolated in the Krasnodar krai of the Russian Federation, demonstrate essentially the same properties as bacteria found in North America, Europe, or Southeast Asia, the spread of antibiotic resistance is clearly a global problem (due to common principles of animal husbandry) and requires internationally established solutions with the involvement of the WHO and UN resources, and cooperation between national governments. Humanity is getting closer to the point of no return, after which even the simplest infections will become untreatable due to the global spread of antibiotic-resistant pathogens. But there is hope that this can still be prevented.

## Figures and Tables

**Table 1:** Morphological and physiological characteristics of the *E. coli* strains.

Characteristic	*E. coli* 533	*E. coli* 546	*E. coli* 923	*E. coli* 933
Serotype	O141:K99	O41:K99	O26:F41	O157:K88
Toxin production	ST-II	RTX α-hemolysin, LT, ST-II	Stx2e, RTX α-hemolysin, AB5, microcin V	Stx2e, RTX α-hemolysin, AB5
Production of hemolysins	E	A, D, E	E	A, D, C, E
Type of hemolysis	β-hemolysis	α-hemolysis	β-hemolysis	α-hemolysis
Colony type on solid media	S-form	S-form	S-form	S-form

**Table 2:** Biochemical characteristics of *E. coli* strains.

Characteristic	*E. coli* 533	*E. coli* 546	*E. coli* 923	*E. coli* 933
H_2_S.	–	–	–	–
IND	+ ^1^	+	+	+
INO	–	–	–	–
VPT	–	–	–	–
LYS	+	+	+	+
ORN	–	+	–	–
ONP	–	–	+	+
URE	–	–	–	–
LAC	–	–	+	+
GLU	+	+	+	+
ADO	–	–	–	–
ARB	+	+	+	+
CEL	–	–	–	–
DUL	–	–	–	–
ESL	–	–	–	–
MAL	–	–	+	+
PHE	–	–	–	–
RAF	+	+	–	–
SCI	–	–	–	–
SOR	+	+	+	+
SUC	+	+	–	–
TRE	+	+	–	–
MAN	+	+	+	+

^1^ “+”—positive reaction; “–”—negative reaction. Production capacity: H_2_S—hydrogen sulfide, IND—indole, INO—inositol, VPT—acetoin. Presence: LYS—lysine decarboxylase, ORN—ornithine decarboxylase, ONP—ß-galactosidase, URE—urease. Fermentation capacity: LAC—lactose, GLU—glucose, ADO—adonitol, ARB—arabinose, CEL—cellobiose, DUL—dulcitol, ESL—esculin, MAL—malonate, PHE—phenylalanine, RAF—raffinose, SCI—Simmons citrate, SOR—sorbitol, SUC—sucrose, TRE—trehalose. MAN—ability to break down mannitol.

**Table 3:** Sensitivity of isolated strains to antibiotics.

Antibiotic	*E. coli* 533		*E. coli* 546		*E. coli* 923		*E. coli* 933	
Aminoglycosides								
Amikacin (AMK)	≤18	R ^1^	≥18	S ^1^	≥18	S	≤18	R
Gentamicin (GEN)	≤17	R	≤17	R	≤17	R	≤17	R
Streptomycin (STR)	≤24	R	≤24	R	≤24	R	≤24	R
Tobramycin (TOB)	≤16	R	≤16	R	≤16	R	≤16	R
Β-lactams								
*Penicillins*								
Amoxicillin (AMC)	≤24	R	≤24	R	≤24	R	≤24	R
Amoxicillin/Clavulanic acid (AMC/CLA)	≤19	R	≤19	R	≤19	R	≤19	R
Ampicillin (AM)	≤14	R	≤14	R	≤14	R	≤14	R
Benzyl penicillin (PEN)	≤30	R	≤30	R	≤30	R	≤30	R
Carbenicillin (CB)	≤22	R	≤22	R	≤22	R	≤22	R
Oxacillin (OX)	≤18	R	≤18	R	≤18	R	≤18	R
Ticarcillin/Clavulanic acid (TIC/CLA)	≥20	S	≥20	S	≥20	S	≥23	S
*Cephalosporins*								
Cefoperazone (CFZ)	≥21	S	≥21	S	≥21	S	≤21	R
Cefotaxime (CTX)	≥20	S	≥20	S	≥20	S	≥20	S
Ceftazidime (CAZ)	≤19	R	≥22	S	≤19	R	≤19	R
Ceftriaxone (CFX)	≤22	R	≤25	R	≤22	R	≤22	R
Cefuroxime (CEF)	≤19	R	≤19	R	≤19	R	≤19	R
*Carbapenems*								
Imipenem (IMN)	≤19	R	≥22	S	≤22	R	≤19	R
Meropenem (MRN)	≥22	S	≥22	S	≥22	S	≥22	S
Glycopeptides								
Vancomycin (VAN)	≤15	R	≤15	R	≤15	R	≤15	R
Lincosamines								
Clindamycin (CLI)	≤15	R	≤15	R	≤15	R	≤15	R
Macrolides								
Azithromycin (AZM)	≤18	R	≤18	R	≤18	R	≤18	R
Clarithromycin (CLR)	≤14	R	≤14	R	≤14	R	≤14	R
Tylosin (TIL)	≤15	R	≤15	R	≤15	R	≤15	R
Erythromycin (ERY)	≤15	R	≤15	R	≤15	R	≤15	R
Nitrofurans								
Furadonin (FUR)	≤21	R	≤21	R	≤21	R	≤21	R
Oxazolidinones								
Linezolid (LZD)	≤25	R	≤25	R	≤25	R	≤25	R
Polypeptides								
Colistin (COL)	≤24	R	≤24	R	≤24	R	≤24	R
Rifampicin								
Rifampicin (RIF)	≤14	R	≤14	R	≤14	R	≤14	R
Sulfonamides/diaminopyrimidines								
Trimethoprim/sulfamethoxazole (TMP/SMZ)	≥14	S	≤11	R	≥14	S	≥14	S
Tetracyclines								
Doxycycline (DOX)	≤16	R	≤16	R	≤16	R	≤16	R
Tetracycline (TET)	≤14	R	≤14	R	≤14	R	≤19	R
Phenicols								
Laevomycetin (chloramphenicol) (LEV)	≤21	R	≤21	R	≤21	R	≤21	R
Florfenicol (FFC)	≤18	R	≤18	R	≤18	R	≤18	R
Fluoroquinolones								
Levofloxacin (LFX)	≤19	R	≤19	R	≤23	R	≤19	R
Norfloxacin (NFX)	≤24	R	≤24	R	≤24	R	≤24	R
Ofloxacin (OFX)	≤22	R	≥24	S	≤24	R	≤24	R
Ciprofloxacin (CIP)	≤22	R	≤25	R	≤22	R	≤22	R
Enrofloxacin (ENR)	≤21	R	≥22	S	≥22	S	≥22	S
Other pharmacological groups								
Fusidic acid (FUS)	≤15	R	≤15	R	≤15	R	≤15	R
Amphotericin (AMT)	≤15	R	≤15	R	≤15	R	≤15	R

^1^ R—resistant to antibacterial drugs (ABDs); S—sensitive to ABDs. Shown numbers represent reference growth inhibition zone diameters (mm) in accordance with EUCAST, Clinical Breakpoint Tables v. 15.0, valid from 1 January 2025.

**Table 4:** Multiple antibiotic resistance index and antimicrobial resistance profile of isolated *E. coli* strains.

Antibiotic Resistance Profile	Strain	Antibiotic Resistance (Number of ABDs)	MAR
AZM, AMK, AMC, AMC/CLA, AM, AMT, PEN, VAN, GEN, DOX, IMN, CB, CLR, CLI, COL, LVC, LFX, LZD, NFX, OX, OFX, RIF, STR, TET, TIL, TOB, FFC, FUS, FUR, CAZ, CFX, CEF, CIP, ENR, ERY	533	35	0.875
AZM, AMC, AMC/CLA, AM, AMT, PEN, VAN, GEN, DOX, CB, CLR, CLI, COL, LVC, LFX, LZD, NFX, OX, RIF, STR, TET, TIL, TOB, TMP/SMZ, FFC, FUS, FUR, CFX, CEF, CIP, ERY	546	31	0.775
AZM, AMC, AMC/CLA, AM, AMT, PEN, VAN, GEN, DOX, IM, CB, CLR, CLI, COL, LVC, LFX, LZD, NFX, OX, OFX, RIF, STR, TET, TIL, TOB, FFC, FUS, FUR, CAZ, CFX, CEF, CIP, ERY	923	33	0.825
AZM, AMK, AMC, AMC/CLA, AM, AMT, PEN, VAN, GEN, DOX, IMN, CB, CLR, CLI, COL, LVC, LFX, LZD, NFX, OX, OFX, RIF, STR, TET, TIL, TOB, FFC, FUS, FUR, CFZ, CAZ, CFX, CEF, CIP, ERY	933	35	0.875

**Table 5:** Draft genome assembly and annotation statistics.

	*E. coli* 533	*E. coli* 933	*E. coli* 546	*E. coli* 923
Completeness, %	100	100	100	99.9
Contamination, %	5.4	2.3	0	6.6
Total number of contigs	961	710	315	1058
Number of contigs (>1000 bp)	466	266	145	347
Total length of contigs (>1000 bp)	5,119,394	5,130,089	5,042,042	5,058,267
N50	16,921	32,352	111,514	24,460
L50	92	42	16	68
Genome coverage	13.7x	15.7x	54.1x	12.8x
GC content, %	50.57	50.29	50.61	50.24
Proteins with functional assignments	5060	4873	4661	4949
Hypothetical proteins	930	861	603	1029
tRNA	80	81	83	92

**Table 6:** Numbers of antibiotic resistance genes and VAGs found in the genomes of *E. coli* isolates.

Database	*E. coli* 533	*E. coli* 933	*E. coli* 546	*E. coli* 923
Antibiotic Resistance (Victors)	4	3	4	3
Antibiotic Resistance (CARD)	90	77	78	82
Antibiotic Resistance (NDARO)	2	1	1	1
Antibiotic Resistance (PATRIC)	68	62	61	68
Drug Target (DrugBank)	413	401	397	415
Drug Target (TTD)	66	63	61	66
Transporter (TCDB)	1024	921	924	942
Virulence Factor (PATRIC_VF)	228	225	217	221
Virulence Factor (VFDB)	101	111	86	109
Virulence Factor (Victors)	256	251	239	250

**Table 7:** AMR and target genes, identified by whole-genome sequencing of *E. coli* isolates, that could participate in antibiotic resistance.

Mechanism of Antibiotic Resistance	Genes	Function
Antibiotic inactivation enzyme	*blaEC* family	β-lactamases, cleave the β-lactam ring of penicillins, cephalosporins, oxime-cephalosporins, and monobactams
Antibiotic resistance gene cluster, cassette, or operon	*marA*, *marB*, *marR*	the operon, which influences multidrug resistance by modulating efflux pumps and porin expression
Antibiotic target in susceptible species	*folA*, *dfr*, *folP*	folic acid biosynthesis, sulfonamide antibiotics (trimethoprim/sulfamethoxazole) target
	*gyrA*	DNA gyrase subunit, primary target of fluoroquinolones
	S12p gene	ribosomal protein, aminoglycoside target data
Antibiotic target protection protein	*bcrC*	quaternary ammonium compound efflux SMR transporter, named after bacitracin
Efflux pump conferring antibiotic resistance	*acrAB-tolC*	a major efflux pump, protects against a wide spectrum of drugs, including β-lactams, tetracyclines, fluoroquinolones, rifamycin, phenicols, etc.
	*acrAD-tolC*	carries aminoglycosides out of the cell
	*acrEF-tolC*	efflux pump, acts against beta-lactams and fluoroquinolones
	*acrZ*	entry into the AcrAB-TolC channel through the inner membrane from the cytoplasm side
	*emrAB-tolC*	MSF pump for fluoroquinolone efflux
	*emrD*	MFS pump, expels amphipathic compounds across the inner membrane
	*emrKY-tolC*	provides resistance to tetracyclines
	*macA*	macrolide export protein, periplasmic MFP in the MacAB-TolC pump
	*macB*	ATPase in MacAB-TolC efflux system
	*mdfA/cmr*	MdfA—efflux of tetracyclines, disinfectants, and antiseptics; Cmr—chloramphenicol resistance
	*mdtABC-tolC*	RND efflux pump, aminocoumarin antibiotics
	*mdtEF-tolC*	RND efflux pump, macrolides, fluoroquinolones, penicillins
	*mdtL*	MFS pump, Na(+)/drug antiporter
	*mdtM*	MFS pump, fluoroquinolones, lincosamide antibiotics, nucleoside antibiotics, amphenicols, disinfectants, and antiseptics
	*sugE*	SMR efflux pump, quaternary ammonium compounds
	*tet(A)*	Resistance to tetracycline by an active tetracycline efflux
	*tolC/opmH*	OpmH is a TolC homolog
Gene conferring resistance via absence	*gidB*	streptomycin resistance due to point mutation in 16S rRNA (guanine527-N7)-methyltransferase gene
Protein altering cell wall charge conferring antibiotic resistance	*gdpD*	glycerolphosphodiesterase
	*pgsA*	phosphatidylglycerophosphate synthetase, mutations in both proteins confer resistance to peptide antibiotic daptomycin

**Table 8:** Sources of the *E. coli* strains studied.

*E. coli* Strain	Date of Isolation	Animal	Organ	Diagnosis
KubGAU B-533 (hereinafter *E. coli* 533)	21 April 2002	Weaned piglet	Mesenteric lymph node	Edema disease
KubGAU B-546 (hereinafter *E. coli* 546)	31 April 2002	10-day-old piglet	Tubular bone	Edema disease
KubGAU B-923 (hereinafter *E. coli* 923)	29 September 2003	Weaned piglet	Spleen	Enteritis
KubGAU B-933 (hereinafter *E. coli* 933)	12 October 2003	10-day-old calf	Mesenteric lymph node; intestine	Enterocolitis

**Table 9:** Deposit of genetic sequences.

Collection	*E. coli* 533	*E. coli* 546	*E. coli* 923	*E. coli* 933
BioSample	SAMN31169425	SAMN31181481	SAMN31181488	SAMN31185483
GenBank	ASM2566039v1	ASM2581757v1	ASM2581758v1	ASM2567444v1
Sequence Read Archive (SRA)	SRS15925162	SRS15925163	SRS15925164	SRS15925165

## Data Availability

The data presented in this study are openly available in BioProject at https://www.ncbi.nlm.nih.gov/bioproject/?term=PRJNA887444 [PRJNA887444], last accessed on 25 December 2025.

## Supplementary Materials

The following supporting information can be downloaded at: https://www.mdpi.com/article/10.3390/ijms27010305/s1.

## Abbreviations

The following abbreviations are used in this manuscript:

ABDs	Antibacterial drugs
AMR	Antimicrobial resistance
MIC	Minimal Inhibitory Concentration
