# Supplementary figures and images for: Genetic Basis of Multiple Antibiotic Resistance of Pathogenic Escherichia coli Strains Isolated from Livestock Complexes in Krasnodar Krai, Russia
Source: Int J Mol Sci. 2025 Dec 27;27(1):305. doi: 10.3390/ijms27010305 (PMC12785339; doi:10.3390/ijms27010305)

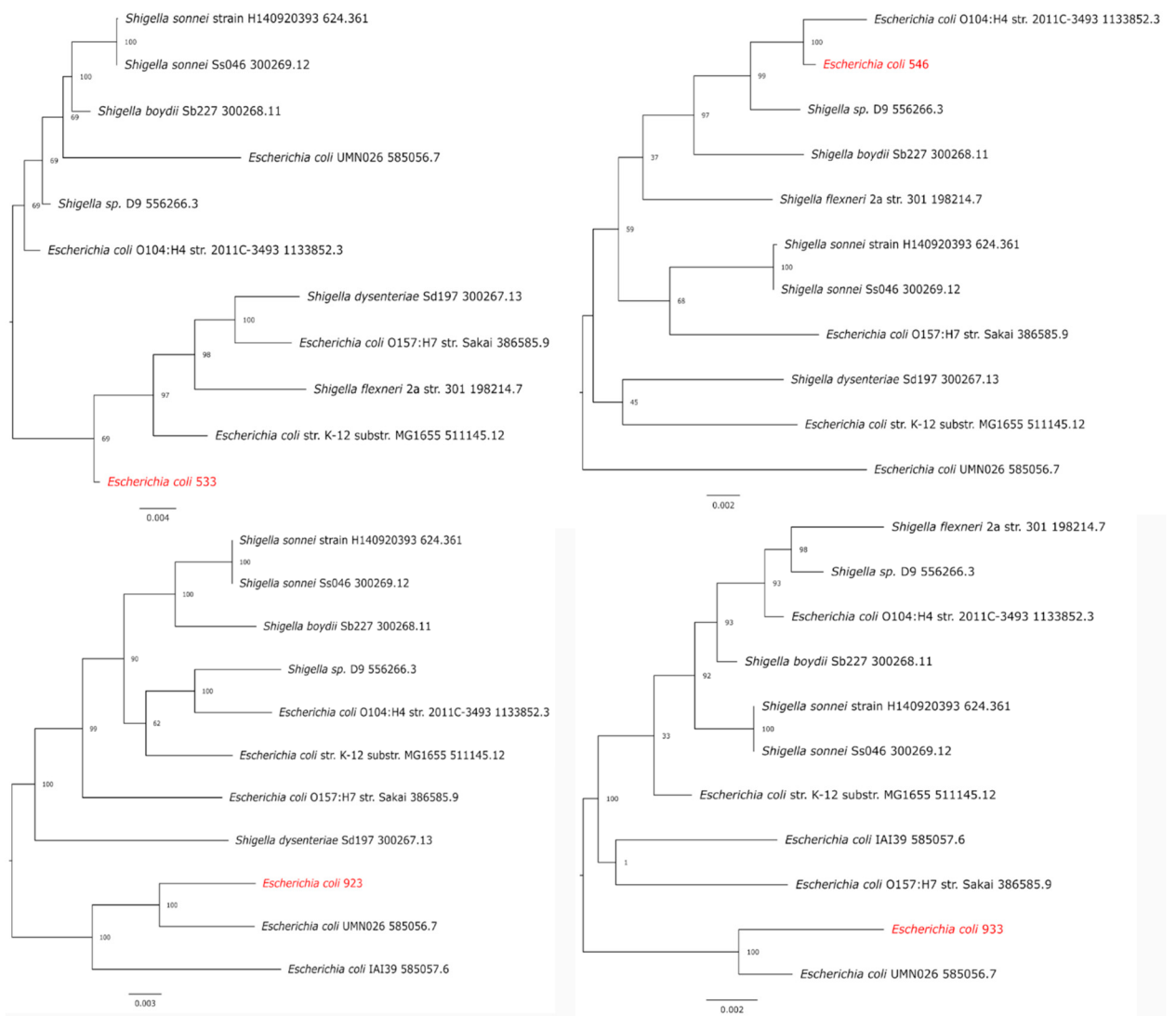

**Figure S1.** Phylogenetic trees for *E. coli* strains 533, 546, 923 and 933 constructed using RAXML.

Supplement: Supplementary file 1 [file ijms-27-00305-s001.zip › Figure S1.pdf]
